# Carbohydrate 19.9 Antigen Serum Levels in Liver Disease

**DOI:** 10.1155/2013/531640

**Published:** 2013-10-27

**Authors:** Gaetano Bertino, Annalisa Maria Ardiri, Giuseppe Stefano Calvagno, Giulia Malaguarnera, Donatella Interlandi, Marco Vacante, Nicoletta Bertino, Francesco Lucca, Roberto Madeddu, Massimo Motta

**Affiliations:** ^1^Department of Medical and Pediatric Sciences, Hepatology Unit, University of Catania, 95123 Catania, Italy; ^2^International Ph.D. Program in Neuropharmacology, University of Catania, 95123 Catania, Italy; ^3^Research Center “The Great Senescence”, University of Catania, 95125 Catania, Italy; ^4^Department of Biomedical Sciences, University of Sassari, 07100 Sassari, Italy

## Abstract

*Background. *Carbohydrate 19.9 antigen (CA19.9) has been used in the diagnosis and followup of gastrointestinal tumours. The aim of this prospective longitudinal study was the evaluation of CA19.9 levels in patients with chronic hepatitis and hepatic cirrhosis hepatitis C virus and B virus correlated. *Materials and Methods*. 180 patients were enrolled, 116 with HCV-related chronic liver disease (48% chronic hepatitis, 52% cirrhosis) and 64 with HBV-related chronic liver disease (86% chronic hepatitis, 14% cirrhosis). Patients with high levels of CA19.9 underwent abdominal ecography, gastroendoscopy, colonoscopy, and abdominal CT scan. *Results.* 51.7% of patients with HCV-related chronic liver disease and 48.4% of those with HBV-related chronic liver disease presented high levels of CA19.9. None was affected by pancreatic or intestinal neoplasia, cholestatic jaundice, or other diseases potentially able to induce Ca19.9 elevations. CA19.9 levels were elevated in 43.3% of HCV chronic hepatitis, in 56.3% of HCV cirrhosis, in 45.1% of HBV chronic hepatitis, and in 58% of HBV cirrhosis. *Conclusions.* CA19.9 commonly increases in the serum of patients with chronic viral hepatitis. Elevation of CA 19.9 is not specific for neoplastic disease and is related to the severity of fibrosis and to the viral aetiology of hepatitis.

## 1. Introduction

CA19.9 is a glycoprotein expressed by several epithelial cancers, as well as in normal pancreatic and biliary duct epithelia, and it is used currently in the diagnosis and followup of gastrointestinal tumours [1, 2]. High levels of CA19.9 have been observed in patients with gastric adenocarcinoma, in colon, biliary duct, and pancreatic carcinomas [3–6]. Patients affected by chronic diseases such as pancreatitis, renal failure, and chronic liver disease showed a significant reduction of CA19.9 specificity [7]. The CA19.9 immuno-reactivity was observed in bile ductules and interlobular bileducts of non-neoplastic areas surrounding hepatocellular carcinoma [8]. 

CA19.9 levels increase in non-neoplastic and organ-specific diseases such as acute and chronic pancreatitis, cholelithiasis, cholecystitis, achalasia, acute hepatitis, hepatic cirrhosis, and respiratory diseases and in systemic diseases such as diabetes mellitus and rheumatic and autoimmune disorders. CA19.9 serum levels can be used as marker for the followup of chronic organ-specific inflammations, such as prostatitis [9–16]. An increase in CA19.9 serum levels has been reported in nonneoplastic liver disease such as simple liver cysts, severe steatosis, autoimmune hepatitis, chronic alcoholic hepatitis, and hepatic cirrhosis [16, 17], where CA19.9 levels seem to correlate with the grade of fibrosis and cholestasis [17]. 

The aim of this study was to evaluate serum CA19.9 levels in a cohort of subjects affected by chronic HCV-correlated (CHC) and HBV-correlated hepatitis (CHB), in order to evaluate whether elevated CA19.9 serum levels can be considered a non casual event and/or could depend on the viral infection (HCV or HBV). We also investigated the correlation between CA19.9 serum levels and the severity of the liver disease.

## 2. Methods and Materials

### 2.1. Patients

Eligible patients for this prospective longitudinal study were those who were 18 years of age or older, were infected by HCV genotype 1b (as determined with the use of the INNO-LiPA assay), and had a quantifiable (>6.90*E* + 07 IU/mL) serum HCV RNA level (as determined by polymerase chain reaction, COBAS AmpliPrep/COBAS TaqMan—ROCHE) and those who were infected by HBV, with positivity to HBsAg (as determined with CLIA technique) and quantifiable HBV DNA serum level (as determined with the use of PCR-based analysis). Both HCV- or HBV-infected populations must had elevated serum alanine transaminase levels and findings on liver biopsy consistent with chronic infection. Cirrhotic patients had to have a Child-Pugh score less than 7 to be eligible. Ineligible patients were those who had other liver diseases, as well as those who were affected by cancer, severe jaundice, pulmonary and renal chronic diseases, prostatic diseases, autoimmune diseases, and diabetes mellitus. None of the patients made excessive use of alcohol (>20 g/die) or hepatotoxic drugs. Study recruitment was performed in observation and respect of Helsinki Declaration. All patients gave their informed consent for the study participation and for each invasive procedure they underwent. All sensitive data were collected and protected in respect of present privacy statements. From November 2007 to January 2009, 180 eligible patients have been enrolled (108 males, 72 females). The entire cohort is split into two groups depending on the viral aetiology: Group 1 contains HCV-infected subjects (74 males, 42 females; mean age 54, range 35–73 years); Group 2 contains HBV-infected subjects (34 males, 30 females; mean age 53, range 34–75 years). Clinical evaluations, hematochemical, virological, instrumental, and histological analysis were performed on these patients. 

### 2.2. Laboratory Analysis

The following serum analyses are reported in details: renal function tests, serum K^+^ and Na^+^, fasting and postprandial glucose, PSA (prostatic specific antigen) serum assay, AST and ALT (aspartate aminotransferase and alanine aminotransferase), *γ*GT (gamma glutamil tranferase), alkaline phosphatase, total, conjugated and unconjugated bilirubin, cholinesterase, serum proteins, prothrombin, fibrinogen, and prothrombin time (PT) analysis were performed. Chemical and physical urine exam was also performed. All patients underwent a complete virological assay for HBV and HCV. HBsAg (hepatitis B surface antigen), anti-HBc IgG (hepatitis B “core” IgG antibody), HBeAg (Hepatitis B “e” antigen), HBeAb (hepatitis B “e” antibody), HBV-DNA (hepatitis B virus DNA), anti-Delta (Delta virus antibody) assays were performed. Genomic analysis for HBV-DNA and HBV genotypes was performed using COBAS AmpliPrep and INNOLIPA Genotyping assay. Anti-HCV antibodies were determined by ELISA (Enzyme-Linked immunosorbent assay ELISA assay-Ortho Diagnostic Systems, Raritan, NJ, USA). HCV-RNA (Hepatitis C Virus RNA) levels were detected by polymerase chain reaction (PCR) of HCV-RNA 5′UTR using COBAS AmpliPrep/COBAS TaqMan (Roche Diagnostics Systems, Branchburg, N.J). HCV viral genotypes were determined by restriction analysis of HCV-RNA 5′ UTR, using Simmonds' classification [18]. Anti-nuclear (ANA), anti-mitochondrial (AMA), anti-smooth muscle (SMA), anti-liver/kidney microsome type 1 (LKM1) auto-antibodies were measured using immunofluorescence assay (IFA) and semi-quantitative ELISA immobilizing enzyme test. Thyroid function was evaluated by levels of TSH (Thyroid-Stimulating Hormone) (WHO 2nd IPR 80/558-ECL), free T3 (free-Triiodothyronine) and free T4 (free-Tetraiodothyronine T4) that were determined by immunoradiometric assay (IRMA) and antibodies against thyroid peroxidase and thyroglobulin were measured by IFA. Serum C3 and C4 assay was performed. Serum levels of iron (n.v. 55–160 mg/dL in men, 45–150 mg/dL in women), saturated transferring (n.v. 2–36 g/L), total transferrin (total iron binding capacity, TIBC n.v. 218–411 mg/dL), unsatured transferrin (unsatured iron binding capacity, UIBC n.v. 110–290 mg/dL) and ferritin (n.v. 18–370 ng/mL) were determined, and saturation of transferrin was calculated as: serum level of iron/TIBC × 100 v.n. 0–40. Genetic testing to identify hemochromatosis HFE gene mutations was performed in order to exclude subjects with hereditary hemochromatosis. CA19.9 measurement was performed in all patients by ECL method Elexis COBAS (n.v. 0.0–39.0 UI/mL) using a commercial kit (CA19.9 diagnostic immunoassay-ROCHE) based on company's instructions. The standard cut-off value (100 UI/mL) grants a sensitivity and specificity of 96% and 90%, respectively. All liver function tests, hematochemical and hormonal measurements, and virological analysis have been executed in the laboratory of our hospital with automated and standardized methods, in conformity to the quality certified standards EN ISO 9001 : 2000. 

### 2.3. Histological Assessment of the Liver

Patients underwent ultrasound-assisted percutaneous biopsy: tissue specimens were obtained with Menghini modified needles (automatic aspiration needle for liver biopsy, ACR 16G, 11 cm, manufactured by Sterylab Srl, Milan; Italy). A Specimen 5 cm long and containing at least 6 portal spaces was considered significant. Histological evaluation of the grade of necroinflammatory activity (grading) and fibrosis (staging) of hepatic tissue was carried out using the METAVIR scoring system [19]. 

Patients who resulted positive to high serum levels of CA19.9 were subjected to perform abdominal and thyroid ecography, thorax radiography, Esophagogastroduodenoscopy (EGD), colonoscopy, and thorax and abdominal spiral TC. Clinical, instrumental, and laboratory investigations allowed to exclude other causes of serum CA19.9 elevation: pancreatic, intestinal, ovarian, mammalian, and prostate tumours, chronic respiratory disease, autoimmune diseases, rheumatic disease, inflammatory bowel diseases, cholestatic jaundice, and hereditary hemochromatosis. Baseline characteristics of the study cohort are shown in Table 1.

## 3. Statistical Analysis

Continuous variables have been presented with mean values ± standard deviation (SD). Dichotomic variations will be expressed as frequencies, and significance was examined by a nonparametric statistical method (Mann-Whitney *U* Test). *P* < 0.05 was considered statistically significant.

## 4. Results

In group 1 (HCV infected subjects), none of patients (0/116; 0%) was found to have METAVIR score F0; 56/116 (48.3%) patients resulted affected by chronic hepatitis (CH, METAVIR F1–F3) and 60/116 (51.7%) by hepatic cirrhosis HCV-correlated (METAVIR F4). 60/116 (51.7%) patients presented high serum levels of CA19.9 (mean value 76.8 UI/mL, SD = 42.3 UI/mL); 26/60 (43.3%) patients with score from F1 to F3; 34/60 (56.7%) with score F4 (cirrhosis). In group 2 (HBV infected subject), all patients were HBeAg negative, anti-HBe positive, and anti-Delta negative; HBV-DNA was detectable in 48/64 (75%) patients. D genotype was found in 39/48 (81.2%) cases, genotype A in 4/48 (8.3%), genotype D + A in 2/48 (4.2%), genotype D + F in 2/48 (4.2%), genotype D + F + A in 1/48 (2.1%). 33/64 (51.7%) patients showed METAVIR F0; 14/64 (21.9%) showed METAVIR F1-F3 (CH); 17/64 (26.6%) showed METAVIR F4 (cirrhosis). In this group, all patients with score ≥ F1 (31/64; 48.4%), showed high serum levels of CA19.9 (mean value 78.8 UI/mL, SD = 41.6 UI/mL). No patients were found with F0 score in group 1 as well as no increase of CA19.9 serum level was observed in patients with METAVIR score F0 in group 2 (33/64; 51.7%). Our results showed that the increase of CA19.9 serum levels in patients with hepatic cirrhosis is statistically significant (*P* < 0.05) in comparison to CA19.9 increase in patients with chronic hepatitis, in both groups 1 and 2 (Tables 1 and 2). Furthermore, our results show a highly significant difference (*P* < 0.001) about the increase of CA19.9 serum levels considering the viral aetiology (HCV versus HBV) of the liver damage when a comparison is made between the overall amount of patients with elevated CA19.9 serum levels in both groups. 

## 5. Discussion

Our data have shown that high levels of CA19.9 are a frequent event in viral chronic hepatitis HCV- and HBV-related diseases, 51.7% and 48.4%, respectively. They often cannot be considered as a sign of neoplastic disease and are statistically correlated with the severity of the disease (*P* < 0.05) and with an increase of the fibrotic process. In fact, elevations of CA19.9 serum levels can be secondary to the necroinflammatory processes, to small bile ducts alterations, to the presence of regeneration nodules, and to the hyper production of raw collagen, which all are typical expression signs of chronic hepatitis progression to liver cirrhosis. 

A number of studies have investigated the clinical utility of CA19.9 in diagnosing pancreatic cancer, cholangiocarcinoma, and other malignancies [20, 21]. Previous studies have shown that serum CA19.9 levels are also elevated in a broad range of benign and malignant conditions, including inflammatory bowel disease, rheumatoid arthritis, pancreatitis, achalasia, heavy tea consumption, Sjogren's syndrome, Hashimoto's thyroiditis, cholangiocarcinoma, colorectal, hepatocellular, esophageal, and lung and ovarian carcinomas [21–23]. 

CA19.9 is a mucinic type glycoprotein that is present only in traces in serum and is normally absent in other tissues such as pulmonary epithelium, perialveolar interstitial space, and liver. It is reasonable that any noxa (viral or toxic) which is able to promote tissue inflammatory damage and, sequentially, reparative features with fibrotic tissue deposition and parenchymal regeneration (as it happens in chronic liver disease, with intra- and interacinar fibrosis, nodular regeneration, and biliary neoductulation) can induce CA19.9 synthesis [24]. CA19.9 immunoreactivity was observed in cell membranes facing biliary canaliculi and in biliary ductules of hepatic bioptic specimens of patients with chronic hepatitis and liver cirrhosis [8]. Immunohistochemical analysis for CA19.9 showed high reactivity in hepatic inflammatory areas, in particular, in bile ductule cells and hepatocytes in ductular metaplasia, suggesting that these cells can be involved in CA19.9 synthesis, and its serum levels increase [8, 25–27]. 

The first outcome of our study is that it is necessary to carefully evaluate the use of CA19.9 as a tumoral marker in the presence of a chronic liver disease, because a high percentage of false positives can occur. Our data also focused on the possibility to use CA19.9 as indirect marker of hepatic fibrosis. 

Some works in the literature report a statistically significant correlation between CA19.9 serum levels and some standard parameters of hepatic function: a positive correlation can be shown with levels of AST, ALT, alkaline phosphatase and bilirubin [17]. Other studies showed a correlation between CA19.9 (alone or together with CA 125) and the grade of hepatic fibrosis. In these studies two groups of patients have been examined, one with a F3-F4 METAVIR score (portal fibrosis without septa, or septal without cirrhosis) and one with a F4 METAVIR score (severe septal fibrosis with cirrhosis). CA19.9 serum levels (alone or together with other serum markers) can be used to individuate patients with severe fibrosis and cirrhosis [28]. 

Transaminases have been the first indirect hepatic fibrosis markers. They have been eventually associated to each other in the aspartate aminotransferase/alanine aminotransferase ratio (AAR) index [28]. Wai and other authors have proposed a further evolution of AAR index, by combining AST to platelet count [aspartate aminotransferase platelet ratio index (APRI)] [29–33]. 

## 6. Conclusion

The increase of CA19.9 serum levels are frequent in chronic viral hepatitis. In our population of patients with viral chronic hepatitis and cirrhosis, CA19.9 serum levels elevation does not indicate a contemporary neoplastic disease, but correlates in a statistically significant way (*P* < 0.05) with the grade of liver fibrosis, appearing to be more evident in patients with higher fibrosis score, thus correlating with the severity of the liver disease. A previous study showed similar results but was conducted on subjects with HCV and did not evaluate subject with HBV [34–36]. A novelty in this study could be represented by the elevation of CA19.9 serum levels that seems to be related to the viral aetiology, showing a high statistical significance when HCV-infected are compared to the HBV-infected patients (*P* < 0.001). This could be explained by a more fibrogenic property of HCV than HBV, but more researches must be conducted to better assess the specific role of the virus for Ca19.9 neosynthesis, while a direct interaction between HCV proteins and hepatic stellate cells (HSCs) may contribute to HCV-induced liver fibrosis. Further investigations may clarify if CA19.9 can assume the role of indirect marker of hepatic fibrosis and be not only as neoplastic biomarker. 

We propose that CA19.9 could be used in the combinations with the other markers already in use, in order to increase the diagnostic accuracy of the available tests, rising both the positive (PPV) and the negative (NPV) predictive values.

## Figures and Tables

**Table 1:** Baseline characteristics of the study cohort.

Characteristic	Group 1	Group 2	
	HCV patients (*N* = 116)	HBV patients (*N* = 64)	
	*N* (%)	*N* (%)	
Sex—*n*° (%)			
Male	74 (63.8)	34 (53.1)	
Female	42 (36.2)	30 (46.9)	
Age (years)	54 ± 2.3	53 ± 2.6	
Race	caucasian	caucasian	
Body-mass index (Kg/m^2^)	26.5 ± 1.0	27.6 ± 1.2	
Blood pressure (sys/dia)—mmHg	128 ± 0.8/84 ± 0.3	132 ± 1.1/81 ± 0.7	
Weight (Kg)	68.5 ± 1.3	71.2 ± 0.6	
AST—IU/L (n.v. 8–18)	63 ± 0.7	73 ± 0.6	
ALT—IU/L (n.v. 8–18)	75 ± 1.2	81 ± 0.8	
*γ*GT—IU/L (n.v. 2–30)	34 ± 1.4	32 ± 1.1	
Cholinesterase—IU/L (n.v. 4900–11900)	5982 ± 102.5	5878 ± 190.3	
Alkaline Phosphatase—IU/L (n.v. 35–100)	86 ± 0.8	89 ± 1.0	
Total bilirubin—mg/dL (n.v. 0.2–1.2)	1.06 ± 0.5	1.07 ± 0.3	
Conjugated bilirubin—mg/dL (n.v. 0.0–0.4)	0.16 ± 0. 2	0.14 ± 0.1	
Viremia			
Mean—log IU/mL	6.40 ± 0.86	6.31 ± 0.92	
Genotype	1b (100)	D	39 (81.2)
		A	4 (8.3)
		D + A	2 (4.2)
		D + F	2 (4.2)
		D + F + A	1 (2.1)
Fasting glucose—mg/dL (n.v. 74–106)	86 ± 1.3	79 ± 1.5	
Serum urea—mg/dL (n.v. 6–40)	42 ± 2.1	39 ± 2.3	
Serum creatinin—mg/dL (n.v. 0.7–1.3)	0.7 ± 0.1	0.6 ± 0.2	
Serum proteins—mg/dL (n.v. 6–8)	7.1 ± 0.6	7.4 ± 0.3	
Fibrinogen—mg/dL (n.v. 200–400)	325 ± 10.3	316 ± 11.5	
Prostatic Specific Antigen—ng/mL (n.v. < 0.4)	1.3 ± 0.7	1.8 ± 0.5	
ANA, AMA, SMA, LKM1	Negative	Negative	
C3-C4—mg/dL	91 ± 1.6	89 ± 1.9	
PLTs count-cell/mm^3^	218 ± 19 × 10^3^	216 ± 21 × 10^3^	
Prothrombin time-sec	10 ± 0.3	9.8 ± 0.2	
Prothrombin-%	112 ± 0.9	104 ± 1.3	
INR—% (n.v. 0.9–1.2)	1.2 ± 0.2	1.1 ± 0.6	
Child-Pugh score	≤5	≤5	
CA19.9—IU/mL (v.n. < 39)	76.8 ± 42.3	78.8 ± 41.6	

Values are expressed as the mean ± SD. The body-mass index is the weight in kilograms divided by the square of the height in meters.

*Viremia stands for quantitative HCV-RNA in Group 1 and HBV-DNA in Group 2 respectively.

**Table 2:** Distribution of patients with elevated serum Ca19.9 according to METAVIR score.

METAVIR*	Group 1	Group 2
	HCV patients (*N* = 60)	HBV patients (*N* = 31)
	*N* (%)	*N* (%)
F1–F3	26 (43.3)^A^	14 (45.2)^B^
F4	34 (56.7)^C^	17 (54.8)^D^

total	60 (51.7)^E^	31 (48.4)^F^

*P* values**: A vs B = ns; C vs D = ns; A vs C ≤0.05, B vs D ≤0.05 and E vs D ≤0.001		

*Liver biopsy specimens were assessed by local pathologist for histology status and reviewed by one expert pathologist, blinded about specimens group assignment.

**P was calculated using a non parametric test (Mann-Whitney *U* test).
